# Investigating the Intermediate Water Feature of Hydrated Titanium Containing Bioactive Glass

**DOI:** 10.3390/ijms22158038

**Published:** 2021-07-27

**Authors:** Mostafa Mabrouk, Hanan H. Beherei, Yukiko Tanaka, Masaru Tanaka

**Affiliations:** 1 Refractories, Ceramics and Building Materials Department, National Research Centre, 33El Bohouth St. (Former EL Tahrir St.), Dokki, Giza P.O. 12622, Egypt; mostafamabrouk.nrc@gmail.com (M.M.); hananh.beherei@gmail.com (H.H.B.); 2 Institute for Materials Chemistry and Engineering, Kyushu University, Fukuoka 812-8582, Japan; yukiko_tanaka@ms.ifoc.kyushu-u.ac.jp

**Keywords:** intermediate water, bioactive glass, Ti-doping, biocompatibility

## Abstract

Intermediate water (IW) in hydrated bioactive glasses remains uninvestigated. We obtained titanium (Ti)-containing bioactive glasses (BGTs) (Ti at 5%, 7.5% and 10% of the glass system) using the sol–gel technique. Their thermal, physicochemical, and morphological properties, before and after Ti-doping, were analysed using DTA, XRD, FTIR, TEM, and SEM accessorised with EDAX, and size distribution and zeta potential surface charges were determined using a NanoZetasizer. The IW in hydrated BGTs was investigated by cooling and heating runs of DSC measurements. Moreover, the mode of death in an osteosarcoma cell line (MG63) was evaluated at different times of exposure to BGT discs. Ti doping had no remarkable effect on the thermal, physicochemical, and morphological properties of BGTs. However, the morphology, size, and charges of BGT nano-powders were slightly changed after inclusion of Ti compared with those of BGT0; for example, the particle size increased with increasing Ti content (from 4–5 to 7–28 nm). The IW content was enhanced in the presence of Ti. The mode of cell death revealed the effect of IW content on the proliferation of cells exposed to BGTs. These findings should help improve the biocompatibility of inorganic biomaterials.

## 1. Introduction

There is an urgent need to improve the properties of bioactive glasses (BGs) to suit all the new applications. This can be achieved by improving the methods of preparation and by adjusting the composition and structure of BGs at the nanoscale. In recent years, studies on glass have demonstrated several restrictions in their medical applications owing to the lack of mechanical properties, as well as high solubility, which disturbs the activity of cells [1]. A key feature of bioactive materials is their ability to form bone-likeapatite on their surfaces in vivo and in vitro. The formation of an apatite layer in the laboratory is affected by many variables, including the starting materials used, pH, concentration of ions, rotation of the soak solution in the laboratory, and composition of the glass and synthesis parameters. Calcium silicate phosphate-based BGs are designed to gradually biodegrade over time and replace the natural host tissue.

Several in vitro and in vivo studies have been conductedto test the bioactivity and cell viability in such systems [2,3,4,5,6,7,8,9], but reports on simple titanium (Ti)-impregnated systems are scarce [5]. In the biomedical field, Ti has a major physiological utility. Because of its biocompatibility (good cell adhesion and viability), Ti is the most comprehensive material for implants used for bone defect augmentation, such as for internal-type dental implants. Special features (enhanced biomineralisation and physicomechanical features) are needed for some applications, and it is preferable to use glasses and/or glass ceramics containing titanium dioxide (TiO_2_) [2,3,4,5]. The role of TiO_2_ in BGs and vitreous ceramics is still under discussion.

Previous studies have shown that the incorporation of TiO_2_ in glasses not only improves their mechanical properties and stability but also increases the non-linear refractive index and ideally modifies the physical properties (density, molar volume, thermal expansion coefficient, glass transition temperature, etc.) [6,7]. By allowing the maintenance of biological activity, the addition of TiO_2_ to glass-based systems leads to the development of biologically compatible and biologically active materials [8,9].

Although various types of materials have been used widely in biomedicine, most biomaterials lack the desired functional properties to interface with biological systems and have not been engineered for optimum performance. Accordingly, there is an increasing demand for solutions to these obstacles in biomedicine. Cellular behaviour can be adjusted through the modulation of several parameters of BG biomaterials. The molecular mechanisms determining the biocompatibility of BGs are complicated and have not been clearly elucidated, despite several theoretical and experimental attempts to explain these mechanisms. Water and protein interactions are recognised as being fundamental to the biological response to contact with polymers [10,11] and an “intermediate water (IW)” concept has been proposed. The presence of IW has been confirmed in hydrated biopolymers (proteins, polysaccharides, and nucleic acids, [DNA and RNA]) and hydrated biocompatible synthetic polymers. It has been hypothesised that polymers possessing the IW property inhibit the direct adhesion of blood cells and proteins to the surface of the polymer. This plays an important role in the compatibility of blood with biocomposites. In this regard, we highlight herein, for the first time, the effect of IW on the development of bioactive glass as an implantable material for bone regeneration. We aimed at linking the physicochemical and morphological properties with the amount and type of bound water in hydrated BG samples along with the presence or absence of Ti in the network of the BG.

## 2. Results and Discussion

### 2.1. Thermal Analysis

**Figure 1:**
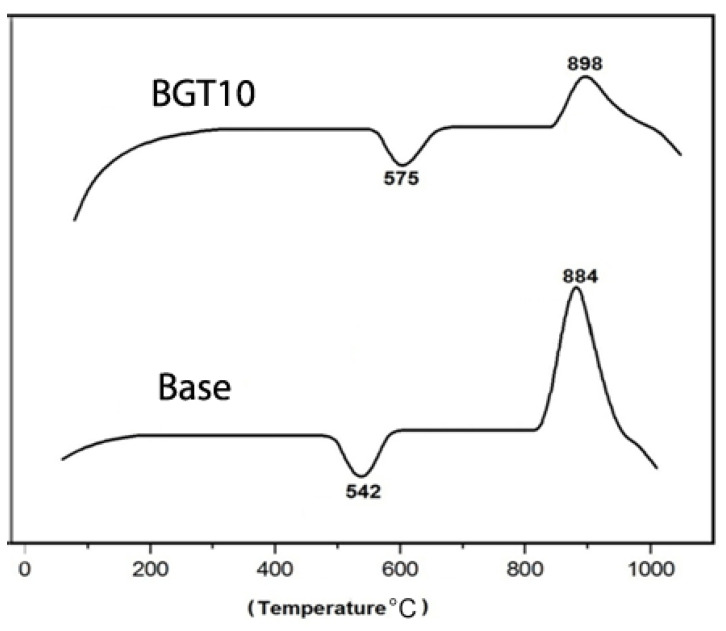
Differential thermal analysis curve of BGT10 with reference to that of native glass (Base).

To determine the effect of doping BG with TiO_2_ on its thermal behaviour, differential thermal analysis (DTA) measurements were conducted. Figure 1 shows the thermal behaviour of BG with higher TiO_2_ content (BGT10) with respect to that of the native glass (Base). To predict the effect of metal doping on the thermal behaviour of glass samples, the glass system without any dopant was compared with the ones with higher dopant percentages, as it was thought to have a more pronounced effect compared to those with lower dopant percentages. It is evident that the presence of TiO_2_ has a remarkable effect on the thermal behaviour, especially on the glass transition (Tg) and crystallisation (Tc) temperatures, of the obtained titanium-containing bioactive glass (BGTs) when compared to that of the BGT0 sample. The presence of TiO_2_ increased the Tg from 542 to 575 °C and Tc from 884 to 898 °C. The significant effect of TiO_2_ in increasing the Tg and Tc temperatures could be explained by the fact that the number of non-bridging oxygen decreased with the increase in the TiO_2_ content in the glass. This increase resulted in an increased temperature of endothermic peaks (i.e., more energy is required to induce crystallisation). Previously, it has been shown that TiO_2_ acts as a flux and a phase-separation inhibitor during the firing regime [10,11]. The glass compositions (weight %) of the prepared samples are listed in Table 1. The Ti-doping percentages were selected based on previous studies in different BG systems including phosphate and silicate glass systems [2,9,11]. These systems were doped with different low (0.0–2.5 molar ratios) and high (up to 10 weight ratio) concentrations; however, remarkable changes in their features were observed in both the cases. Thus, we decided to combine both low and high Ti doping in the present study to determine their effect on the final features of the achieved BGs.

### 2.2. Physicochemical Characterisation

#### 2.2.1. X-ray Diffraction (XRD) Analysis

**Figure 2:**
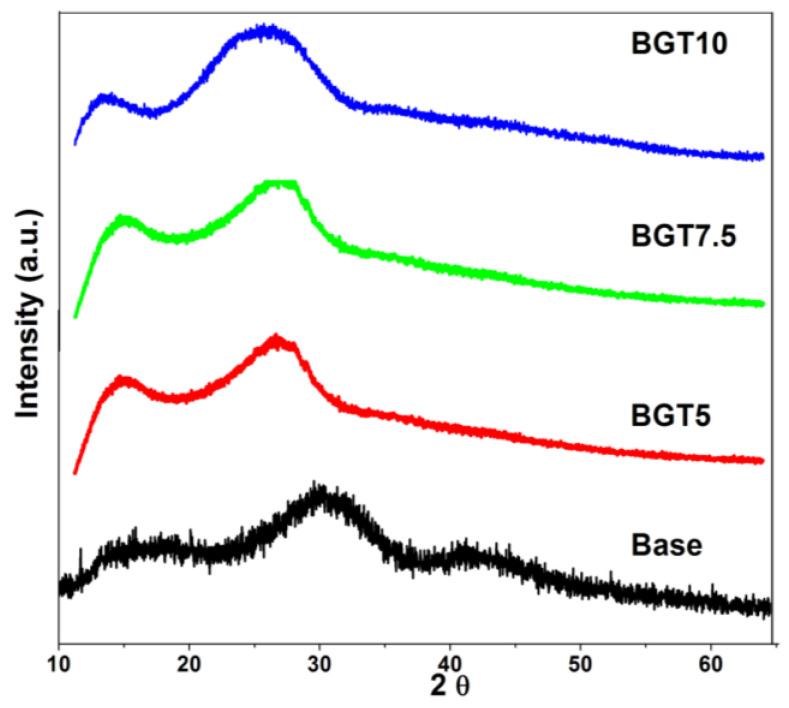
X-ray diffraction curves of the prepared glasses doped with TiO_2_.

The amorphous nature of the prepared glass samples before and after doping with Ti was confirmed based on the XRD curves presented in Figure 2. It is obvious that the inclusion of TiO_2_ within the glass network did not alter the physical nature of these samples, as all of them exhibited amorphous curves. However, the presence and quantity of TiO_2_ were associated with notable changes in the position of the glassy hump region. The base system showed a hump in the region of 25–35 2θ, BGT5 had its main hump in the range of 22–30 2θ, BGT7.5 exhibited a hump in the region of 10–25 2θ, and BGT10 showed a main hump in the range of 18–32 2θ. Same results have been reported for the inclusion of different transition metals in several studies; however, in most of these studies, no remarkable changes were observed in the amorphous nature of glass [12,13,14].

#### 2.2.2. Fourier-Transform Infrared Spectroscopy (FTIR) Analysis

**Figure 3:**
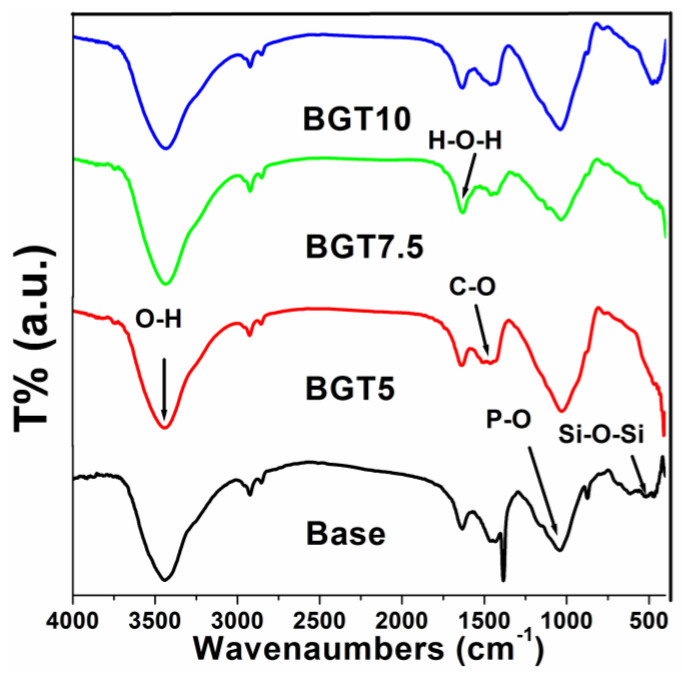
Fourier-transform infrared spectroscopy spectra of the prepared glass doped with TiO_2_.

The functional groups of the prepared glass samples before and after doping with Ti were determined from the FTIR spectra shown in Figure 3. It is clear that the incorporation of TiO_2_ into the silicate-based glass used in the present study has no remarkable effect on the chemical integrity of these samples, as all of them possessed several functional groups related to the base BG. In particular, a band corresponding to the Si-O-Si bending vibration mode at 498 cm^−1^ and the Si-O-Si symmetric stretching vibration of the bridging oxygen between the tetrahedral, which was observed at 780 cm^−1^, were detected [15,16]. These two bands confirm the presence of SiO_2_, the main component of the glass system, as a network former. Moreover, the presence of phosphate groups was also recorded by the band corresponding to the P-O stretching mode in the 1043–1024 cm^−1^ region [17]. The presence of Ti within the glass network was confirmed for the Ti-O vibration band recorded at 635 cm^−1^ [18]. This was also indirectly confirmed by the detection of both asymmetric and symmetric stretching vibrations of the carboxylic group, which can be identified at 1530 and 1440 cm^−1^, indicating Ti as a bidentate ligand [19]. Furthermore, the bending H-O-H functional group was recorded at 1620 cm^−1^ and the O-H stretching vibrations of the water group that were detected at 3500 cm^−1^ were noted, and their intestines were decreased when compared to those for the Base BG. These two bands confirm the formation of hydrogen bonds between the Ti atoms and OH groups [20,21].

### 2.3. Morphological Properties

#### 2.3.1. Transmission Electron Microscopy (TEM)

**Figure 4:**
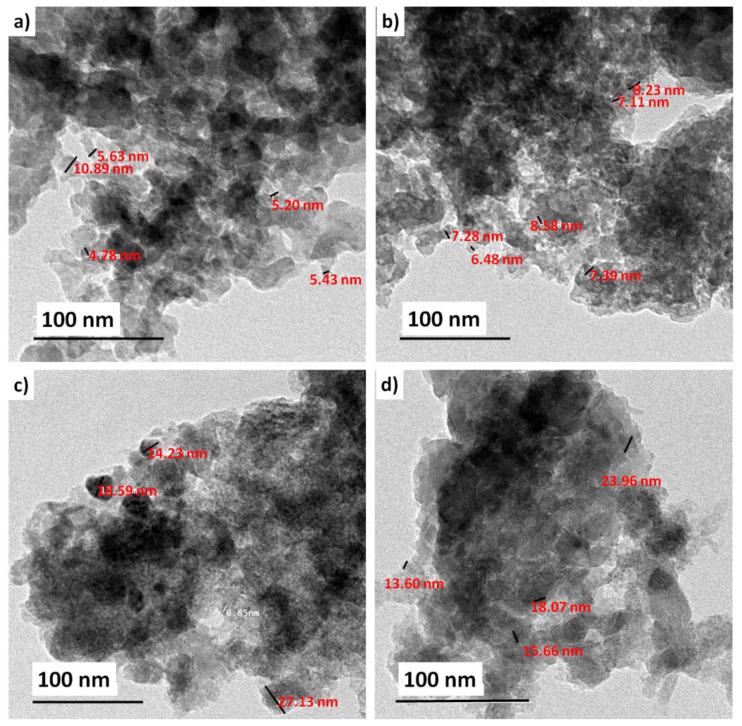
Transmission electron micrographs of TiO_2_-doped glass with reference to the native glass; (**a**) Base, (**b**) BGT5, (**c**) BGT7.5 and (**d**) BGT10.

The morphology and size of the dispersed native BG, as well as of the TiO_2_-doped ones, were captured by TEM, and the images are illustrated in Figure 4. Generally, the obtained pure glass (BGT0) particles were found to be on the nanoscale (4–5 nm) (Figure 4a). Furthermore, relative gradual increases in the particle sizes were noted with increasing TiO_2_ content in the 7–28 nm range (Figure 4b–d). Titanium is expected to play different roles in the glass formation process; the first scenario is that it acts as a network modifier, which is not very common at higher concentrations, as in the present case. The best scenario would be that Ti acts as a network former, resulting in double condensation, which in turn increases the particle size [22,23,24,25]. However, the morphology of the obtained BGs before and after the inclusion of Ti showed an agglomerated appearance, typical of BG nanoparticles [26,27].

#### 2.3.2. Scanning Electron Microscopy (SEM)

**Figure 5:**
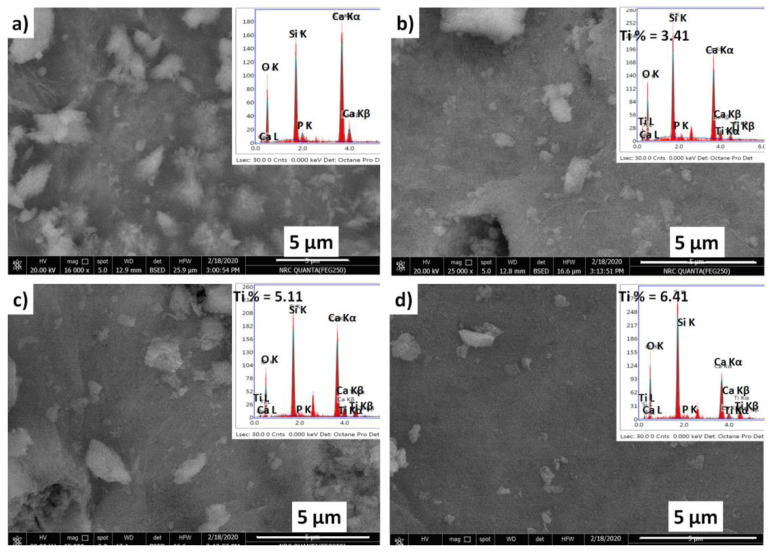
Scanning electron micrographs and corresponding energy-dispersive X-ray spectroscopy spectra of TiO_2_-doped glasses with reference to the native glass; (**a**) BGT0, (**b**) BGT5, (**c**) BGT7.5 and (**d**) BGT10.

The bulk morphology and elemental composition of BGT s were recorded using a SEM-EDAX instrument, the results of which are shown in Figure 5. All the prepared samples exhibited very amorphous surfaces composed of very fine particles; no particlefusion was detected in the micrographs, which suggests the absence of crystallised nanoparticles. This result is in agreement with the XRD results and confirms the amorphous nature of the obtained glasses. Moreover, the elemental composition of the native glass was confirmed by its corresponding energy-dispersive X-ray spectroscopy (EDAX) chart; the observed elements were Ca, Si and P, which matched the main components of the hypothesised system. In addition, the inclusion of TiO_2_ in the glass network was confirmed, and its measured content was relatively close to the hypothesised one. Recently, the presence of inorganic elements in biomaterials has been correlated with in vitro uptake of water and the final biocompatibility features of biomaterials in several studies [28,29,30]. It is believed that the capacity of biomaterials to absorb water, which is associated with their expanding capacity, is pivotal for the stability of the biomaterial structure and transportation of cell supplements and metabolites present in biomaterial substrates.

#### 2.3.3. Size Distribution and Zeta Potential: Dynamic Light Scattering (DLS) Zetasizer

**Figure 6:**
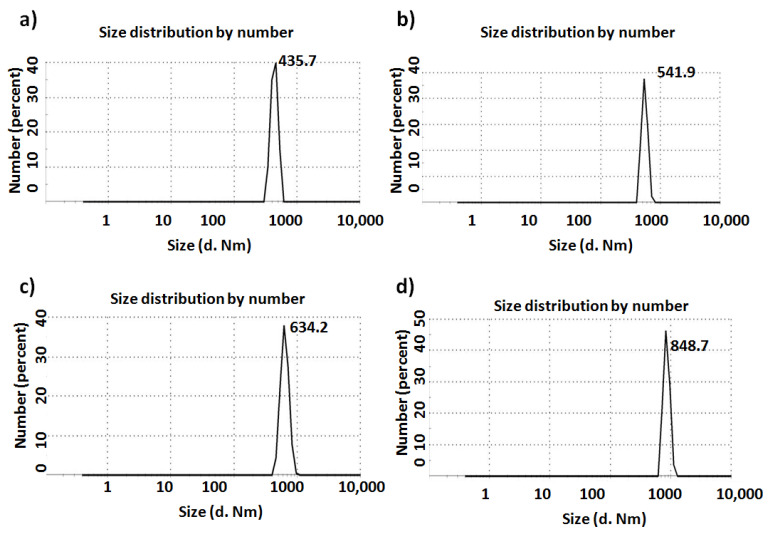
Particle size of (**a**) BGT0, (**b**) BGT5, (**c**) BGT7.5 and (**d**) BGT10 determined by Zetasizer.

The size and surface charges of the glass particles in the presence and absence of TiO_2_ were determined using a DLS Zetasizer. The particle sizes that were recorded for the BGTs exhibited an increase when compared to those for the native glass (see Figure 6). Particularly, native glass (BGT0) showed a higher intensity of size distribution of 435.7nm, whereas glasses incorporated with Ti demonstrated size distributions of 541.9 (BGT5), 634.2 (BGT7.5), and 848.7 (BGT10), respectively. Importantly, DLS revealed higher particle sizes for all the BGT samples, in contrast to the TEM results. This might be because of the hydrophilic characteristics of the fabricated BGTs, which brought about adherence of water and resulted in agglomeration of the nanopowders [31]. This notion can be explained by the difference in sample preparation and working principle of each instrument. However, the two strategies revealed that the sizes of the fabricated nanoparticles are in the nanoscale in either scattered or agglomerated structures. The agglomeration features proved for the BGTs developed in this study also support their potential ability for water absorption in aqueous media, such as cell culture medium and extracellular fluids [32,33].

**Figure 7:**
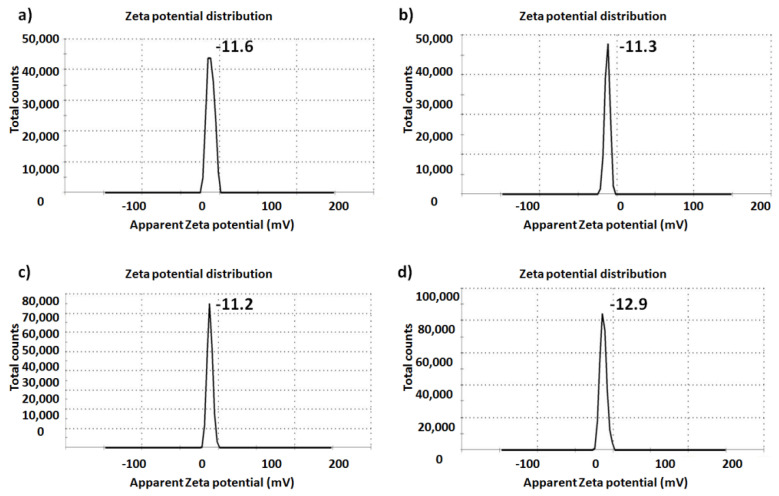
Particlesurface charges of (**a**) Base, (**b**) BGT5, (**c**) BGT7.5 and (**d**) BGT10 determined by Zetasizer.

The zeta potential of the fabricated glasses before and after TiO_2_ inclusion in the glass network is shown in Figure 7. It is worth highlighting that the presence of Ti did not significantly change the surface charge of the fabricated glasses. The zeta potential values were generally negative; the negative charge on the surface of any biomaterial enhances its interactions with biological systems, especially with cells, as previously reported [34,35].

### 2.4. Assessment of Water Content

The type of interfacial water within a material surface is hypothesised to demonstrate an important task in the formation and behaviour of materials in aqueous media, including biological environments. For example, the biocompatibility features of a material, such as protein adsorption on the surface in biological medium, are believed to be highly influenced by the water (especially intermediate water) content of the sample. Therefore, it is very important to consider protein molecules in the hydration state of any material with potential application in biological systems [36,37]. Additionally, it was observed that several components existing in biological media have the ability to alter the activity of water and the hydration state [38]. The water present in the hydrated structure of biomaterials can be classified into three types: free water (scarcely bound water), freezing bound water (intermediate water/loosely bound water), and non-freezing bound water (tightly bound water) [39]. Intermediate water (IW)has been reported to be responsible for determining the interactions (biocompatibility) between cells and materials. Furthermore, the quantity of IW was confirmed to increase with an increase in the inorganic component in polymer materials [10,40]. Based on this fact, BG samples are thought to demonstrate similar advantages as they consist of inorganic elements in oxide network systems. Therefore, the IWs of the fabricated BGT samples in the hydration state were determined using differential scanning calorimetry (DSC).

**Figure 8:**
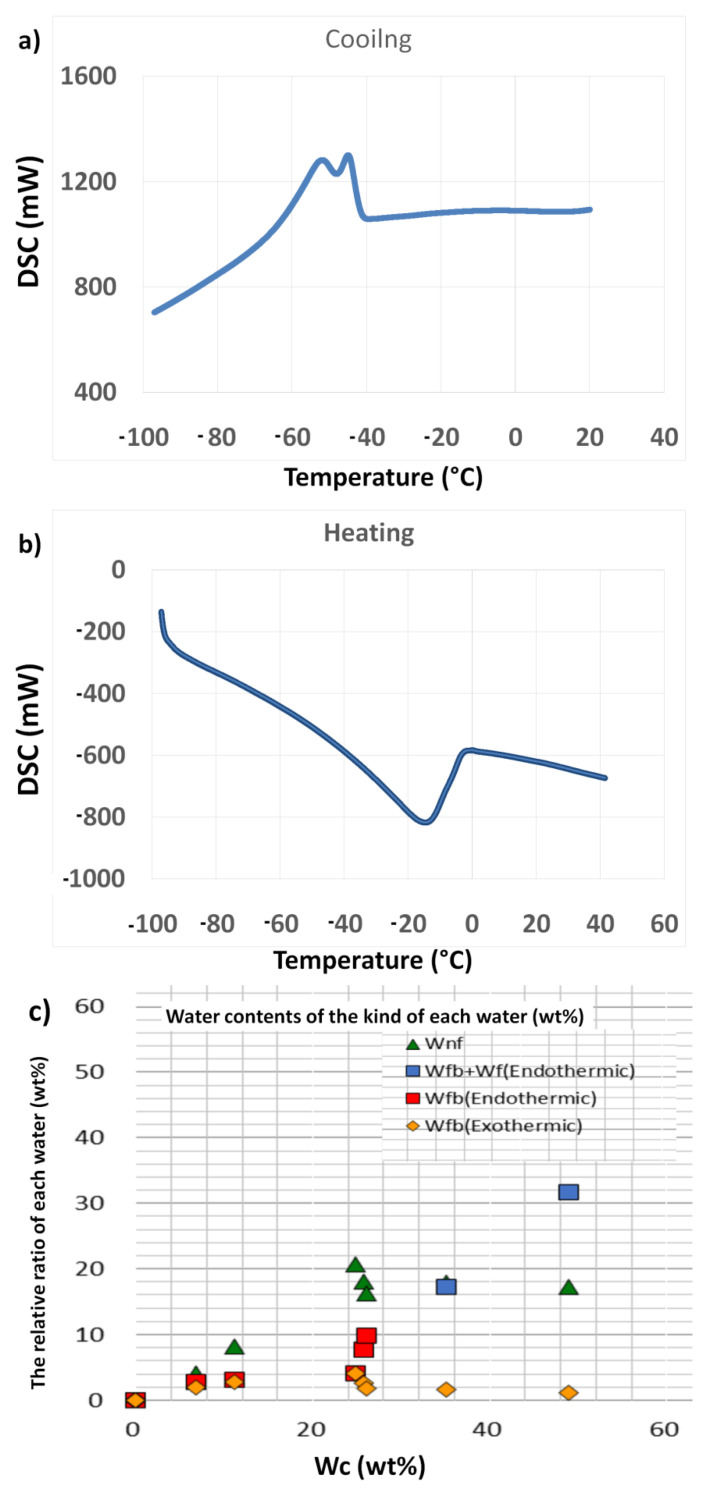
Water content of the BGT0 sample determined by differential scanning calorimetry under (**a**) cooling and (**b**) heating conditions; (**c**) content of each type of water (Wnf = non-freezing water; Wfb = freezing bound water, and Wf = freezing water).

**Figure 9:**
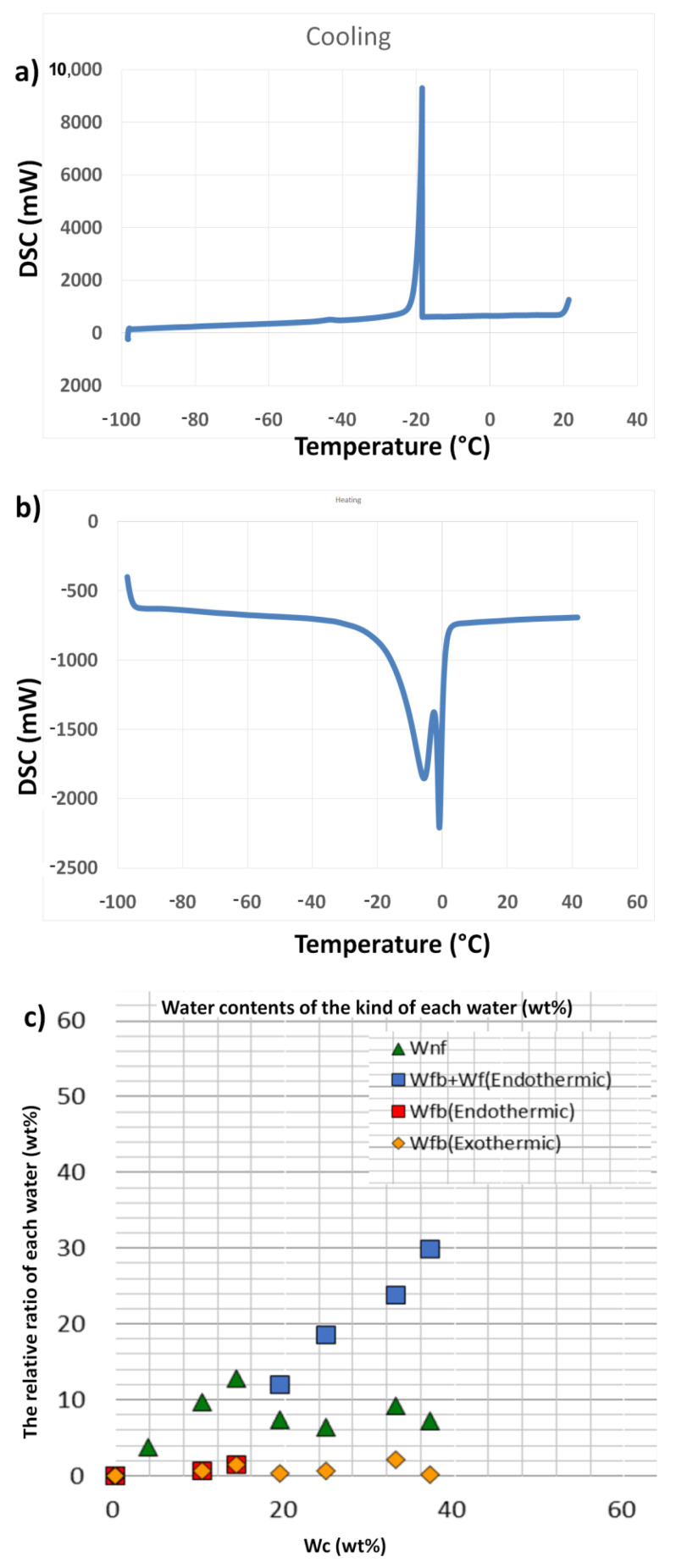
Water content of BGT10 sample determined by differential scanning calorimetry under (**a**) cooling and (**b**) heating conditions; (**c**) water content of each type of water (Wnf = non-freezing water; Wfb = freezing bound water, and Wf = freezing water).

DSC data obtained by cooling and heating runs were used for thermal analysis of two BGT samples (Base glass [BGT0] and Ti-doped glass [BG10]), as shown in Figure 8 and Figure 9 and Supplementary Figures S1 and S2. The cooling scan from 40 to −100 °C of the BGT0 samples, with a cooling rate of 5 °C/min, is shown in Figure 8a,c. Figure 8a shows the exothermic peaks at different temperatures. First, there were no peaks for the dry sample (water content (Wc) = 0 wt %), which indicates that the non-freezing bound water was only present in BGT0 and no peaks for IW or free water were observed. There was a weak broad exothermic peak of hydrated BGT0 sample in the cooling run at a Wc of 6.7 wt % and a temperature of −45 °C for freezing bound water (IW). A broad exothermic peak of hydrated BGT0 sample in the cooling run at a Wc of 11.2 wt % around −43 °C was observed. This peak represents the crystallisation of IW and shifts to the high-temperature side with increasing water content. In Figure 8a, two broad exothermic peaks were observed for hydrated BGT0 sample in the cooling run at a Wc of 24.7 wt % around −45 and −43 °C for crystallisation of IW, which indicates an increase in the amount of IW. It also shows two exothermic peaks; one broad exothermic peak of hydrated BGT0 sample was observed in the cooling curve at a Wc of 25.7 wt % at −45 °C for crystallization of IW and a very sharp exothermic peak was observed at approximately −25 °C, which was also attributed to IW close to free water. This suggests that the transition of the water crystallising phase to an amorphous phase occurred. Significantly, a slight decrease in IW (at −45 °C) was noted when the Wc exceeded 24.7 wt %. It was also noted that with an increase in Wc over 24.7 wt %, the intensity of the IW close to free water (at −25 °C) increased significantly with the shifting of the peaks to higher temperatures.

Figure 8b shows the heating run for −100 to 40 °C forBGT0 sample at a heating rate of 5 °C/min. No peaks were recorded for the dry BGT0 sample, indicating the absence of IW or free water. However, small broad melting peaks attributed to the melting of ice were recognized for hydrated BGT0 sample with Wc values of 6.7, 11.2 and 24.7 around −15 to −4 °C. The DSC measurements revealed different hydration structure s of the BGT0 and BGT10 samples, as listed in Table 2.

These broad peaks were enhanced starting from Wc of 25.7 wt % around −5 °C, and their intensity increased with the increasing water content. The DSC heating run of hydrated BGT0 sample with Wc of 48.9 wt % exhibited a very sharp peak around −2 °C related to IW; however, the quantity of free water was noted above 0 °C, which indicates the presence of bulk water. Further analysis enabled us to assess the quantity of each water type related to the water content in various proportions, as shown in Figure 8c. We confirmed higher proportions of bound-freezing water, especially IW, at various endothermic and exothermic peaks.

Moreover, the thermal behaviour of hydrated BGT10 sample was investigated by cooling and heating DSC runs over a temperature ranging from −100 to 40 °C and Wc ranging from 3.7 to 37 wt %, as illustrated in Figure 9a,b. DSC cooling runs of the hydrated BGT10 sample (Figure 9a) showed no peaks at Wc ranging from 3.7 to 14.2 wt % within the cooling range of 40 to −100 °C. This suggests that the existence of Ti in the glass network delays the occurrence of hydration states at lower content of water; thus, BG becomes more hydrophobic in the presence of Ti. Previous reports have indicated better biocompatibility of biomaterials with improved hydrophobicity [41,42].

The exothermic sharp peak below −21 °C at Wc of 19.3 wt %, which is assigned to IW close to free water, was observed. With increasing Wc up to 24.8 wt %, the exothermic peak shifted toward lower temperature around −22 °C, which was assigned to the crystallisation of IW close to free water. With an increase in Wc to 33 wt %, the exothermic peak shifted again to −20 °C. The cooling run of hydrated BGT10 sample with Wc 33 wt % revealed an exothermic peak at −17 °C, which is attributed to free water.

The endothermic peaks observed in the DSC heating run from −100 to 40 °C are shown in Figure 9b. BGT10 samples hydrated with Wc of 3.7 to 14.2 wt % showed no endothermic peaks, which is consistent with the results of cooling runs and supports the increased tendency towards hydrophobicity. The DSC cooling run of the hydrated BGT10 sample demonstrated two endothermic peaks at approximately −2 and 5 °C. With increasing Wc in the hydrated BGT10 sample to 33 and 37.1 wt %, the endothermic peaks shifted to higher temperatures and the peaks became sharper. This implies that the presence of Ti increased the quantity of IW, especially freezing-bound water, in general, with higher content of water. After determining the proportion of each type of water and their amount recorded at different Wc percentages (Figure 9c), it is suggested that bound-freezing water increases with an increase in Wc. It can be concluded that the amounts of intermediate and non-freezing bound waters are influenced by the Ti doping of BG and the total content of water. We have previously reported similar results for hydroxyapatite substituted with magnesium ions [43].

### 2.5. Death Mode of Cells

**Figure 10:**
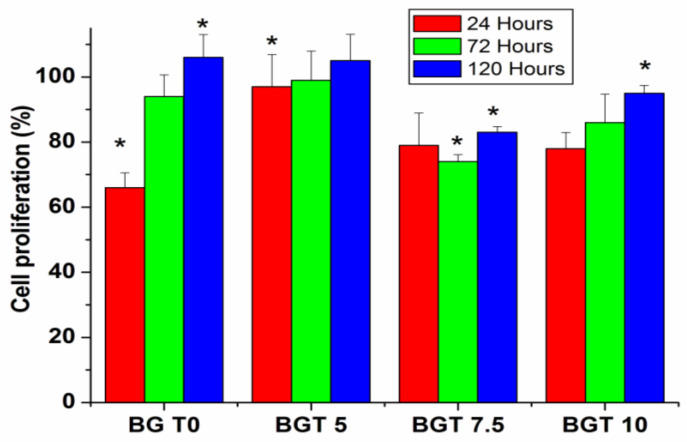
Cell proliferation (%) measured after exposing MG63 cells to bioactive glass discs for different intervals of time. Significant results are marked with “*”.

**Figure 11:**
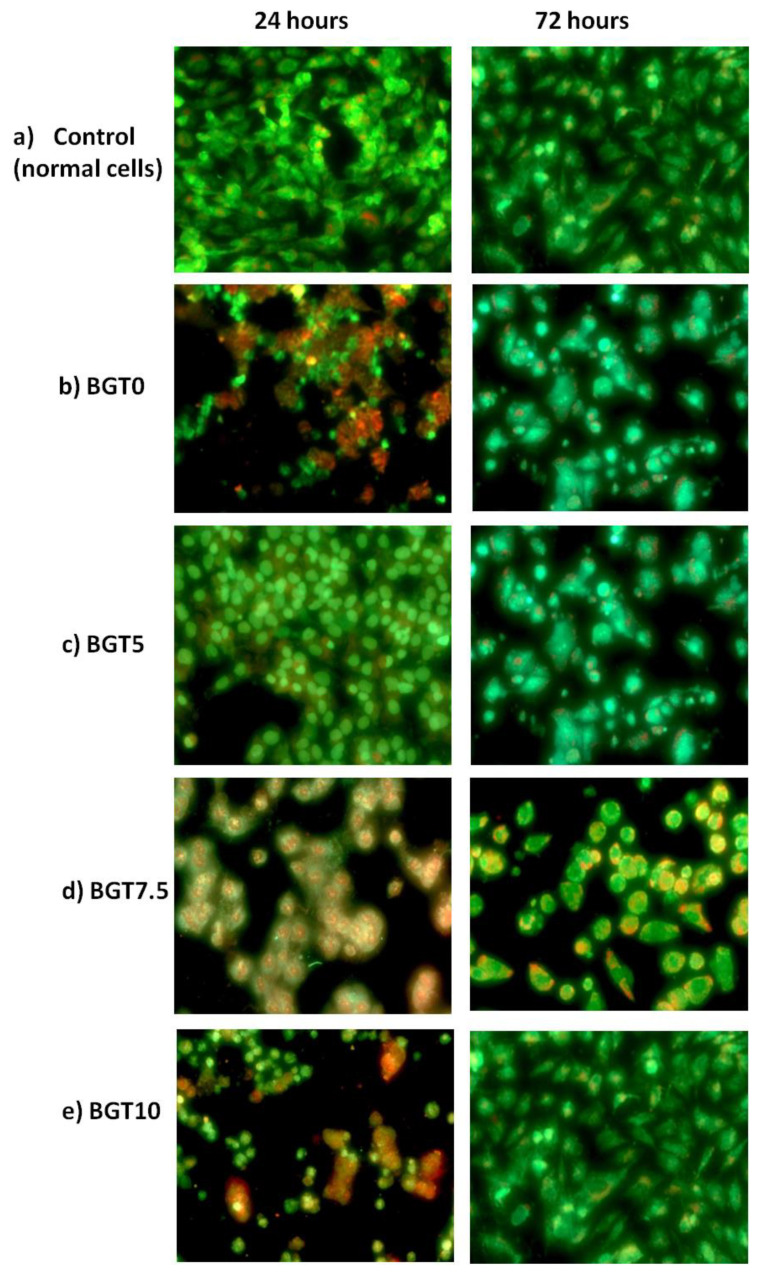
Images (magnification = 20×) were recorded using a fluorescence camera after 24 and 72 h exposure of MG63 cells to different glass types. (**a**) control, (**b**) BGT0, (**c**) BGT5, (**d**) BGT7.5 and (**e**) BGT10.

The mode of death in MG63 cells exposed to each glass sample for different time intervals (24, 72 and 120 h) was evaluated. The cell proliferation (%) is shown in Figure 10. Notably, after 24 hexposure, the viable cell count decreased to 63–78% compared to that of normal cells for all the samples except for BGT5, which showed a very little decrease (97%).The percentage of cells undergoing necrosis was minimal (6–16%) compared to that of apoptosing cells (16–21%). Vital (green), early apoptotic (bright green to yellow), late apoptotic (orange), and necrotic (red) cells very observed. This suggests a lower death rate caused by BGT samples. The BGT5 sample maintained cell viability at 72 (99%) and 120 h (109%). For the other glass samples, the cell viability increased as the exposure time increased, and they demonstrated decreased percentages of necrotic cell death (2–10%) and stable percentages of apoptic death (14–20%). This could be explained by the fact that the cells surviving after 24 h adapted to the glass environment and continued to proliferate under the new conditions. These results were confirmed by images recorded after 24 and 72 h (Figure 11).

It is obvious that only BGT5 possessed relative cell viability when compared to the control, as shown in Figure 11c. After 24 h, cells begin to adapt to the glass environment and continue their growth under the new condition, as shown in Figure 11. These results suggest the biocompatibility of the investigated BG samples in the present study. Based on the findings of water content within the glass samples, the presence of different elements, such as SiO_2_, as the main component of BG and TiO_2_ as the dopant material within the BG networks plays an important role in increasing the intensity of the IW if the samples are hydrated. If the same hydration conditions were provided to the BG samples (cell culture medium), the content of IW is also expected to increase. This, in turn, helps cells to survive and continue proliferation with time. This concept was recently proved for several types of polymers, especially for PMEA and its derivatives [44,45,46,47,48,49].

## 3. Materials and Methods

### 3.1. Glass Preparation Method

A co-solvent with 300 mL each of distilled water and ethyl alcohol was used for the hydrolysis of a predetermined volume of tetraethyl orthosilicate (TEOS), which was added under acidic conditions (drops of concentrated hydrochloric acid [HCl]). This solution was stirred for 30 min before the addition of calcium nitrate tetrahydrate. After 30 min, triethyl phosphate (TEP) was added to the above solution and stirred for another 30 min at room temperature until the mixture appeared homogenous and transparent. For Ti doping, the previous steps were followed by the addition of titanium isopropyl (C_12_H_28_O_4_Ti) as a precursor of TiO_2_. The obtained gel was placed in a furnace at 70 °C overnight to dry and further calcinated at 550 °C for 2 h.

### 3.2. Characterisation Techniques

Several characterisation techniques were applied to the BG system (Ti-containing glass), as shown in Table 2. The following sections describe the characterisation of the glass system.

### 3.3. Thermal Analysis

Differential thermal analysis was used to determine the firing temperature at which the glass was prepared. In addition, the influence of TiO_2_ inclusion within the network of the glass system on the thermal behaviour of TiO_2_-doped BG was evaluated. BG fine powder (10 mg) was fired under atmospheric heating conditions in a platinum crucible. The heating regime from room temperature to 1000 °C at a heating rate of 5 °C/min was followed. The heating was done in a SDT Q600 V20.9 Build 20machine (TA Instruments, New Castle, DE, USA).

### 3.4. Physicochemical Characterisations

#### 3.4.1. XRD Analysis

TiO_2_-doped glass powders with reference to their native glass system were scanned using an AXS D8 ADVANCE X-ray diffraction (XRD) system (Bruker, Karlsruhe, Germany). The XRD instrument was accessorised with a Cu-Kα irradiation Ni-filter, 40KV, and 40mA. This technique was utilised to evaluate the effect of the crystallinity/amorphous nature of the investigated samples.

#### 3.4.2. FTIR Analysis

The chemical composition of the designed TiO_2_-doped glass powders with reference to their native glass system was investigated using a Fourier transform infrared (FT-IR) spectrophotometer (model FT/IR-6100 type A, Jasco, minzen, Germany) in the 400–4000 cm^−1^ range. Prior to examination, the glass sample powders were mixed with KBr powder at a ratio of 2:100.

### 3.5. Morphological Properties

#### 3.5.1. TEM Analysis

AJEM2100 high-resolution transmission electron microscope (TEM-HR), (JEOL, South Dakota, Japan) was used to determine the particle size and morphology of the prepared BG. A few millilitres of BG dispersions before and after Ti doping were prepared. Thereafter, copper grids were immersed in the prepared suspensions and allowed to dry at room temperature for examination using the TEM system.

#### 3.5.2. SEM Imaging

The morphology and elemental analysis of the developed BGs before and after Ti doping were analysed by scanning electron microscopy coupled with energy-dispersive X-ray spectroscopy (EDX) (JEOL JXA-840A electron probe micro-analyzer) at 15 kV. Stainless steel stubs covered with carbon tape were used to monitor the glass samples. Prior to sample screening using SEM, the samples were covered with a thin film of gold.

#### 3.5.3. Size Distribution and Zeta Potential: DLS Zetasizer

A Zetasizer Nano ZS (Malvern Instruments, Worcestershire, UK) equipped with a 633nm laser was used to record electrophoretic measurements. A microfilter (0.22 µm) was utilised to filter each sample suspension prior to the measurements. The filtered samples were loaded onto a disposable capillary cell (DTS1060, Malvern). The cells loaded with samples were then flushed before reloading, and measurement s were made after the second loading. The obtained data were analysed using the software provided with the instrument employing the Smoluchowski equation to determine the particle size and zeta potential.

### 3.6. Assessment of the Water Content

Purified water was utilised to hydrate the glass samples (3–4 mg) prior to DSC measurements. The DSC measurement started with the cooling of the sample, at a rate of 5 °C/min, in the temperature range of 25 to −100 °C. The cooling was followed by a constant temperature at −100 °C for 5 min. This was followed by heating to 50 °C, at a rate of 5 °C/min, under a nitrogen purge flow. Known parameters, such as pure water crystallisation and melting temperatures of approximately −17 and 0 °C, respectively, could be acquired through the entire procedure together with the melting enthalpy of pure water (334 J/g). To ensure that the hydrated sample reached a constant weight, it was dried at 110 °C using a vacuum dryer. Intermediate water (cold crystallisation) is observed in the cooling and heating stages as the main factor that explains the biocompatibility of the sample. The crystallised IW melted at temperatures below 0 °C. As non-freezing water is a part of the chemical structure of the glass sample, it does not crystallise below 0 °C, even at −100 °C, because of a strong interaction with the glass sample. Freezing (free) water melted at 0 °C during heating.

### 3.7. Death Mode of Cells

MG-63 cells (1 × 10^4^ cells) were cultivated on cell culture slides (SPL, Seoul, Korea) with discs of BGT0, BGT5, 7.5 and BGT10, individually, and then dissected after 1, 3 and 5 days with the BG discs. Slides were washed with phosphate-buffered saline (PBS) and stained with ethidium bromide/acridine orange as the fluorophore (EB/AO; 100 µg/mL of each in 100 µL PBS) for 10 min in the dark and then visualised under a fluorescence camera (20× magnification) (Axio Imager Z2, Carl Zeiss Microscopy GmbH, Jena, Germany) with a fluorescent camera (AxioCam MRc3 S/N 4299, Carl Zeiss Microscopy GmbH) and ZEN 11 blue software for image analysis(Carl Zeiss Microscopy GmbH). The proportions of viable, early apoptotic, late apoptotic and necrotic cells were determined for each platform fixation at spans of 1 day and contrasted with those for the control cells (grown under the same experimental conditions without exposure to the investigated BGs.

## 4. Conclusions

The preparation and characterisation of BGTs linked to their IW and assessment of their biocompatibilities are described for the first time. The typical features of the BGTs were not influenced by Ti doping, as indicated by the DTA, XRD, and FTIR results. Minor changes in morphological features of these nanoparticles with increasing Ti-doping percentage were noted. Notable changes in the intensity of the IW content were detected by DSC measurements in the presence of Ti doping. The mode of cell death revealed a correlation between the existence and content of IW taken up by the glass samples and cell survival and proliferation rates. It is believed that adjusting the composition of inorganic materials, such as BGs, would control the presence of IW and its content. This, in turn, will assist in enhancing the final biocompatibility properties. In the near future, our team will try to apply the concept of IW for different glass systems along with different dopant elements. In addition, this concept also needs to be investigated for polymer/BG composites.

## Figures and Tables

**Table 1:** Composition (weight %) of glass doped with titanium.

Sample	Glass Composition (wt %)			
	CaO	P_2_O_5_	SiO_2_	TiO_2_
BGT0	45	5	50	---
BGT5	40	5	50	5
BGT7.5	37.5	5	50	7.5
BGT10	35	5	50	10

**Table 2:** Hydration structure analysis of BGT0 and BGT10 samples determined by differential scanning calorimetry measurements.

Sample	Maximum Intermediate Water (wt %)	Maxium Non-Freezing Water (wt %)	Intermediate Water + Free Water (wt %)	Equilibrium Water Content (wt %)
BGT0	9.8	20.6	17.1	48
BGT10	1.4	12.8	11.9	33

## Data Availability

Data will be available upon request.

## Supplementary Materials

The following are available online at https://www.mdpi.com/article/10.3390/ijms22158038/s1, Figure S1: water content determined by DSC of BGT0 sample (a) cooling and (b) heating conditions; (c) water content of each type of water; Figure S2: water content determined by DSC of BGT10 sample (a) cooling and (b) heating conditions; (c) water content of each type of water.
