# Supplementary material for: Investigating the Intermediate Water Feature of Hydrated Titanium Containing Bioactive Glass
Source: Int J Mol Sci. 2021 Jul 27;22(15):8038. doi: 10.3390/ijms22158038 (PMC8348002; doi:10.3390/ijms22158038)
Supplement: Supplementary file 1 [file ijms-22-08038-s001.zip › ijms-1292496-supplementary.pdf]

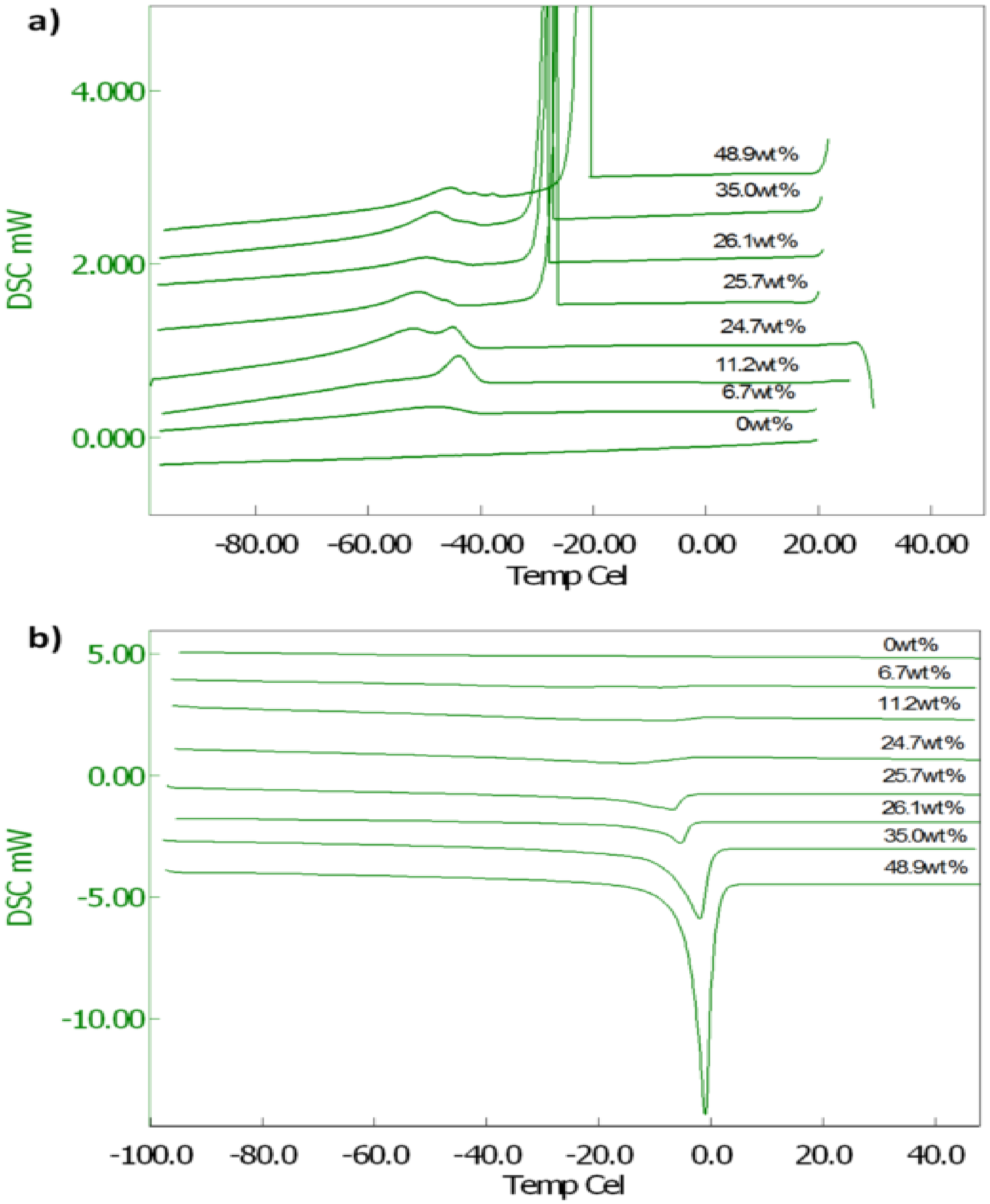

Figure S1. water content determined by DSC of BGT0 sample (**a**) cooling and (**b**) heating conditions; (**c**) water content of each type of water.

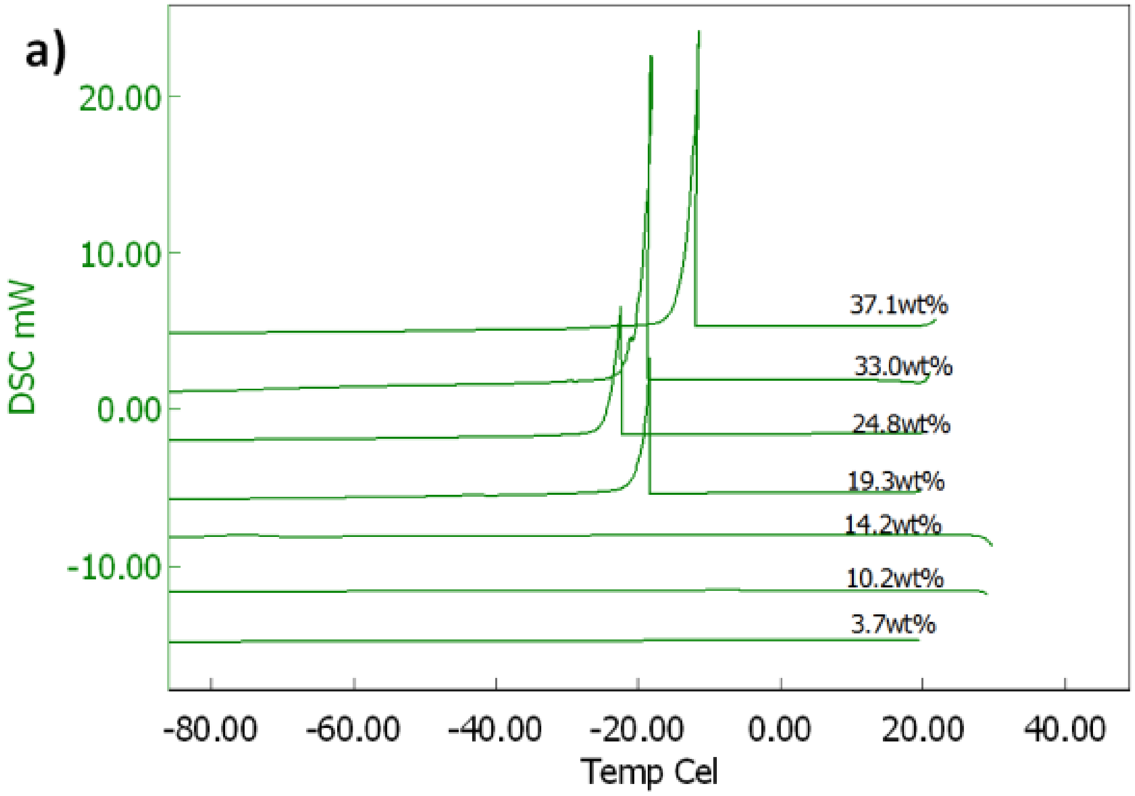

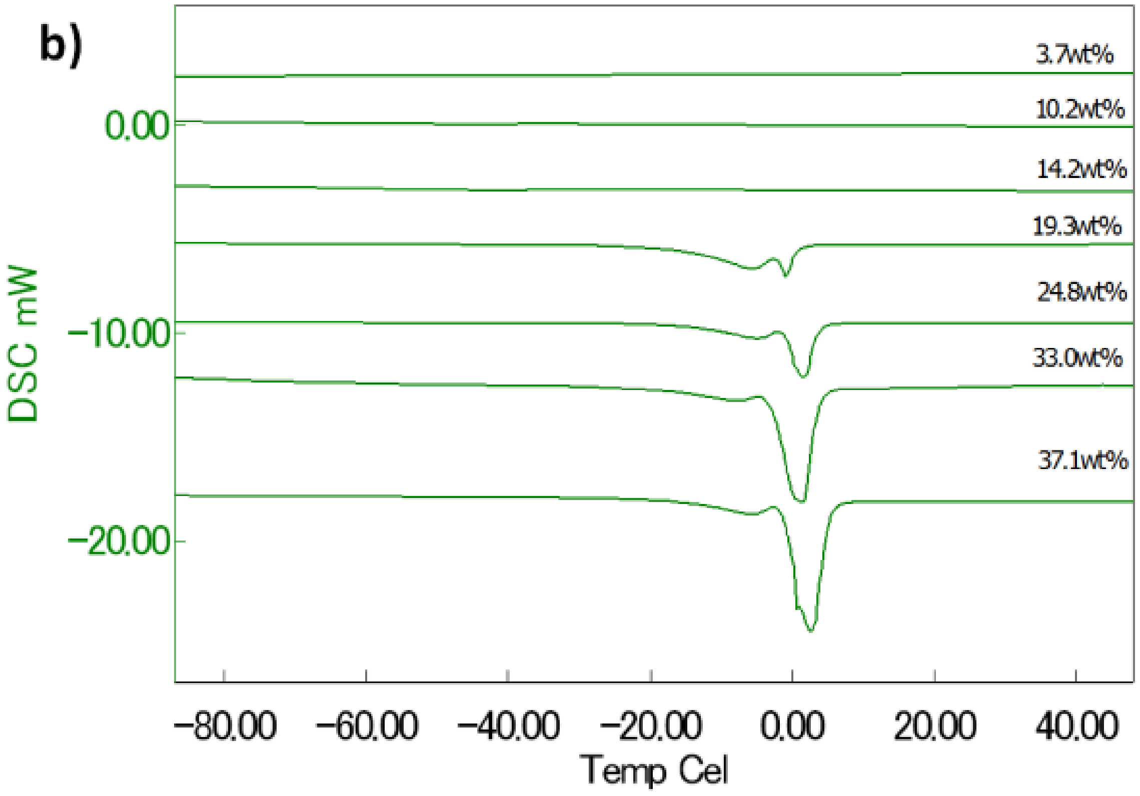

Figure S2. water content determined by DSC of BGT10 sample (**a**) cooling and (**b**) heating conditions; (**c**) water content of each type of water.
